# Self-financing students in private medical schools

**DOI:** 10.3352/jeehp.2012.9.4

**Published:** 2012-01-30

**Authors:** P. Ravi Shankar

**Affiliations:** Department of Medical Education, KIST Medical College, Lalitpur, Nepal.

Dear editor

I read with great interest the comments of the author in the letter titled 'Equity or equality in medical education.' I agree with the author about curriculum impact assessments (CIA) having not concluded that one curriculum is fit for all. I also agree with the author that medical graduates should have a basic set of skills depending on their country, region and community. Many educators believe that a uniform curriculum is needed to develop a common set of competencies. While this may seem logical and straightforward to many, objective evidence to support this premise may be lacking. A major problem in medical education is that research-based evidence to support decisions is often lacking.

Globally, there is a move towards defining competencies in medical education [1]. Generic objectives for community-based undergraduate medical education have been defined [2] and validated through a survey of medical educators in developing nations [3]. Also with health science education fellowships like the two-year program offered by the Foundation for Advancement of International Medical Education and Research (FAIMER) [4] and Master's in Health Professions Education programs in the region, a critical mass of educators is being developed who can drive curriculum change forward. I strongly feel competencies and broad objectives of the curriculum should be defined at a national or even regional level and that universities and medical schools should have the freedom to develop curricula to attain these objectives.

In developing countries in South Asia a number of private medical schools have opened in recent years, and in these schools the agenda of students receives high priority as the major source of funding, for these schools is through student fees [5]. The contribution of the government to infrastructure development and day-to-day running of private medical schools is limited. I agree with the author that medical schools must be socially accountable, but the accountability of private schools may be different from government ones and may have to be worked out through consensus and based on objective evidence. Self-financing students in private schools pay a large amount as tuition fees and recovering this large investment is a major priority for many students. Working in developed nations is seen as a major means towards return of investment. An article published in 2007 showed that the highest number of doctors in the South Asia region migrate from India followed by Pakistan, Sri Lanka, Bangladesh, and Nepal [6]. A large number of students whom the author has taught have migrated to developed nations. I agree with the author that global health or international health is an important priority for medical education. Three structural themes have been defined for global health education, and these are the burden of global disease, travelers' health, and immigrant/refugee health. The international health course at the University of Adelaide, Australia aligns with these three themes [7]. International health electives (IHEs) contribute to the development of social accountability among medical students from developed nations [8]. Medical schools in developing countries benefit from IHEs by the opportunity for the institution/country to earn foreign exchange and also through an increased supply of resources [9]. Networks are also built at both personal and institutional levels.

I am not in agreement with the author that developing countries like Nepal cannot afford to offer even a few electives to students. In Nepal and many other South Asian countries, medical education is being primarily financed by the students themselves. While it may be true that this money is ultimately derived from the country or community, the immediate source of financing are the students' families. In this situation, I feel schools can offer electives to further develop the skills of students in specific areas. Medical councils in developing nations including the Medical Council of India are re-examining the role of electives in the medical curriculum. I had offered medical humanities as a voluntary module to interested students at Pokhara, Nepal [10]. The FAIMER fellowship program requires candidates to develop and implement a curriculum innovation project, and many of these are offered as an 'elective' to interested students. There are a number of health science educators who have completed or are pursuing FAIMER fellowships in India, Nepal, and other countries, and students have benefited from the programs offered. The cost of offering an elective may vary according to the subject area and has not been scientifically studied to the best of the author's knowledge.

Private medical schools, the dominant force in South Asia and Nepal, have three customers. The first and the most important are their students; the others are the patients and the community. I agree that private schools should have a social responsibility. However, this may differ compared to government schools. The impact of privatization in medical and health science education is South Asia is an important area for research [11]. Most private schools in South Asia are set up as for-profit enterprises, and investment and resources are likely to be channeled to areas which provide the maximum returns.
